# News and Coming Events

**Published:** 1908-06-13

**Authors:** 


### News and Coming Events.

The trustees of Smith's (Kensington Estate) Charity have awarded the Hospital for Sick Children, Great Ormond Street, a donation of £500.

The King will place a commemoration stone in King Edward VII.'s Hospital and Dispensary for Windsor, Eton, and District, on Monday, June 22, at 11.30. The building will take the place of the present Windsor and Eton Royal Dispensary and Infirmary.

The Hunterian Society has decided to award a silver medal annually for the best essay by a general practitioner embodying the results of original observations. Intending candidates can obtain full particulars from the senior honorary secretary, Dr. W. Langdon Brown, 37a, Finsbury Square, London, E.C.

The International Surgical Society will hold its second Congress at Brussels this year, from September 21 to 26. The questions proposed for discussion are: (1) Cancer, (2) the surgery of the liver, (3) anæsthesia, (4) the surgery of the pancreas, (5) hernia. A special feature of the Congress is to be a cancer exhibition.

A return by the Registrar-General, Dublin, giving statistics showing the number of deaths in Ireland due to alcohol and tuberculosis for the twelve months ended December 31 has been issued as a Parliamentary paper, [135]. The number of deaths from all forms of tuberculous disease was returned as 11,677.

The Duchess of Albany, attended by Lady Evelyn Moreton, presided on June 4 at the Royal Waterloo Hospital for Children and Women over a meeting of the Working Committee of Ladies' Association connected with that hospital. Last month the Ladies' Association was instrumental in obtaining a donation of £1,000 towards the general maintenance.

At a meeting of the Council of the Pharmaceutical Society of Great Britain, held on June 3, Mr. J. Rymer Young, of Warrington, was re-elected President, it being the unanimous wish of the members of the Council that Mr. Young should retain his position in view of the Society's attitude regarding the proposals of the Government to amend the law relating to the sale of poisons now before Parliament.

The Society for the Destruction of Vermin has appointed a special committee to inquire into the existing laws and regulations concerning noxious insects and animals, to agitate for their more efficient and extended application, and to propose such changes as may be necessary to meet requirements in the light of modern knowledge, especially with regard to the all-important *rôle* played by vermin in the dissemination of disease germs communicable to man and domestic animals.

The French Medical Association known as the E.M.I. proposes to visit England in July and to stay in London during the week beginning July 12. The Franco-British Exhibition will be visited, and also some of the principal hospitals, including more particularly St. Bartholomew's and its new out-patient department. The staff of this hospital will give a reception in honour of the visitors, and an address will be delivered by Professor Landouzy in the Great Hall. Dr. Leonard Williams, together with Dr. Leonard Mark and Mr. Macleod Yearsley, are organising an informal dinner to be held at the Exhibition, and are otherwise making arrangements for the success of the visit.

The annual dinner of the past and present members of the Medical School of the University of Oxford will be held in University College on Saturday, June 20. This year the meeting will take the form of a farewell dinner to Dr. Gustav Mann, who is leaving Oxford to enter on his new duties as Professor of Physiology at New Orleans, U.S.A. The secretary of the dinner is Mr. C. F. Beevor, Magdalen College.

In connection with the Pilkington Cancer Research Foundation of the University of Manchester, three lectures on cancer will be delivered by Dr. C. Powell White on Wednesdays, beginning on June 17 at 4.30 p.m. They will be illustrated by microscopical preparation, museum specimens, and lantern slides, projected by the epidiascope. Members of the medical profession and students of medicine are invited to attend.

The 36th annual meeting of the Provident Surgical Appliance Society, of which Lord Derby is President, was held on May 28 at the offices, 12 Finsbury Circus, Mr. E. Chalk, chairman of the board of management, presiding. During the year between 8,000 and 9,000 surgical appliances have been supplied to the necessitous poor, making a total of 192,000 appliances since the establishment of the society in 1872.

Upon the recommendation of the Secretary for Scotland, the King has approved the appointment of Professor Samson Gemmell, M.D., Professor of Clinical Medicine in the University of Glasgow, as Professor of the Practice of Medicine in that University, in the place of the late Sir T. M'Call Anderson; and of Mr. Francis Mitchell Caird, F.R.C.S., Lecturer on Clinical Surgery in the Royal Infirmary, Edinburgh, as Regius Professor of Clinical Surgery in the University of Edinburgh, in the place of the late Professor Annandale.

H.R.H. the Prince of Wales has contributed £500 to the capital account of the King Edward's Hospital Fund for London, to be invested in such manner as seems best to the Finance Committee. His Royal Highness has been prompted to forward this additional donation by the recent munificent gift of Lord Mount Stephen to the fund, and by the suggestion made by him that the friends of the fund should join in raising such a sum as would enable it to distribute £150,000 a year to the hospitals of London. The following sums, in addition to the Prince of Wales's donation, have been received for this object:—Anonymous, £1,000; Lord Revelstoke, £500; Mr. Frederick M. Fry, £250; Sir Horace Marshall, £500; A Friend, £500.

The marble bust of the late Mr. George Herring will be unveiled at the Mansion House on Monday, June 15, in the presence of the constituents of the Metropolitan Hospital Sunday Fund, the Lord Mayor presiding. The presentation will be made by the Hon. Sydney Holland (one of Mr. Herring's executors); and Bishop Lawrence, of Boston, Massachusetts, and Mr. Henry Morris, President of the Royal College of Surgeons, will address the meeting in support of the objects of the fund. Mr. George Herring bequeathed the residue of his fortune, amounting to about £700,000, to the Hospital Sunday Fund. For eight years before his death he contributed from £10,000 to £12,000 annually, in the last five years adding 5s. in the pound to the amount collected in places of worship. Over a million and a half sterling has been awarded to hospitals and institutions in the thirty-five years of the existence of the Metropolitan Hospital Sunday Fund.

Mr. A. E. Thomas, now of the Royal Free Hospital, London, was presented, on June 3, at the Town Hall, Gravesend, with a silver tea and coffee service subscribed for by the Committee of Management, Hon. Medical Staff, and Ladies' Association of the Gravesend Hospital in appreciation of his eight years' service as secretary to that institution.

In connection with the proposed Putney Hospital (Chester Bequest) a grand bazaar will be held in the house and grounds of The Home Field, Putney Hill, S.W. (by permission of the Council of the Girls' Public Day School Trust, Limited), on Wednesday, Thursday and Friday, June 24, 25 and 26, 1908. H.R.H. the Duchess of Albany will perform the opening ceremony. A souvenir book entitled "'Twixt Heath and River" is being prepared under the editorship of Mrs. Furley Smith, and will be sold for the benefit of the Putney Hospital Fund.

The arrangements for the Garden Fête, which H.R.H. the Princess Louise, Duchess of Argyll, will open on July 7, at the Royal Botanical Gardens, in aid of the Charing Cross Hospital, are progressing satisfactorily. Miss Italia Conti is arranging scenes from "As You Like It" as a woodland masque with special dances and glees. There is also to be a café chantant during tea. Several of the tea tables will be named after popular plays now running, and will be presided over by performers in these plays. Many well-known artistes have already promised to assist in the fête. Tickets can now be obtained at the usual agents and of the Secretary at the Hospital.

The first business meeting of the National Union of Public Health Authorities was held on May 28 at the Caxton Hall, Westminster. The Union was formed some few months ago. The chairman, Alderman Newton, of Newcastle-on-Tyne, congratulated the members upon the excellent progress which had already been made in bringing the Union and its objects before the Public Health authorities of the country. They looked forward, he said, to forming one great union of Public Health workers. The draft constitution was then submitted, showing that the object of the Union was to secure harmony and uniformity of action among Public Health authorities for the purpose of obtaining certain necessary sanitary reforms, of encouraging the study of hygiene, and of educating public opinion as to the importance of health work generally.

We regret to announce the death of Deputy-Surgeon-General Sir James Arthur Hanbury, K.C.B., F.R.C.S., which occurred on June 3 at Bournemouth. Sir James was born in 1832, and entered the medical service of the Army on completing his professional education. He took the degree of M.B. of the University of Dublin in 1853. He served with distinction in China, India, and America, was principal medical officer of a division during the Afghan campaigns of 1878-9 and 1879-80, and was prinicpal medical officer under Lord Roberts on the occasion of the memorable march from Kabul to Kandahar, for which service he received the C.B. In 1882 he was chosen to accompany Lord Wolesley as principal medical officer of the Egyptian expedition, and on its close was created a K.C.B. He was principal medical officer at Gibraltar in 1887 and 1888, and surgeon-general of the forces in the Madras Presidency from the latter year until 1892, when he retired. Sir James Hanbury received the honorary Fellowship of the Royal College of Surgeons in Ireland in 1883, and that of the English College in 1887.

H.R.H. Princess Alexander of Teck will open the extension of the Nurses' Home of Queen Charlotte's Hospital on Tuesday, June 30. The extension will accommodate an additional 35 nurses. The new building also comprises a residential college for students, with accommodation for 16 students and qualified practitioners. A sum of £10,000 is still needed to open the extension free of debt, and donations towards this amount are earnestly asked for.

### General Medical Council.

The proceedings of the summer session of the General Medical Council were concluded on June 3. After a prolonged hearing judgment was given in the case of Mr. John P. Nicolas, M.R.C.S.Eng., L.R.C.P.Lond., who had been summoned to appear before the Council on the charge that he had systematically canvassed in Catford for patients, by personal visits to patients of Mr. T. W. Atkinson and to others, and that in the course of such canvass he had disparaged the qualifications and skill of Mr. Atkinson and extolled his own.

During the course of the hearing Mr. Hemming, solicitor for the accused practitioner, raised a large number of objections and complaints with regard to the conduct of the proceedings against his client. Before the Council gave its decision Mr. Lushington, the Council's legal assessor, said he desired to state in public that every preliminary step before the notice of inquiry was issued up to the first hearing of the case was taken upon the advice of the legal advisers, and, in their opinion, in strict accordance with the standing orders of the Council. If there was any slip in the procedure which had caused substantial injustice to the accused practitioner, there was a remedy open to him by law. The Council deliberated in private. On the public being re-admitted, the President, addressing the accused practitioner, said: "The Council have judged you to have been guilty of infamous conduct in a professional respect, and have directed the registrar to erase from the register the name of John Papa Nicolas." Mr. Hemming applied that the registrar be directed to erase the name in a month's time to give him an opportunity in the interval of applying to the High Court for a writ of *certiorari*. The President replied that the application could be made to the office in the usual way.

Dr. Mackay brought up a report from the Education Committee regarding the receipt from the Privy Council of a letter enclosing a copy resolution, adopted by the members of the Medico-Legal Society and others on March 24 last, urging legislation with a view to making the administration of anæsthetics illegal except by duly registered medical practitioners, and stating that the Lord President would be glad to be favoured with any observations the Council might have to offer thereon. The Committee recommended that a reply be forwarded stating that the Council had consistently endeavoured to safeguard the interests of the public in matters relating to the practice of medicine, and they would, therefore, willingly support any measure which had as its object to render illegal the practice, save by duly qualified and registered medical practitioners, of any department of medicine and surgery, including the administration of anæsthetics; that the Council had within the last year issued a "recommendation" to the licensing bodies that a course of study in the administration of anæsthetics should be included in the curriculum, and that they had reason to believe that the "recommendation" had been already given effect to by many of the bodies. The recommendation was adopted. This concluded the proceedings of the Session.

